# The effect of continuous positive airway pressure therapy on early atherosclerosis in patients with severe obstructive sleep apnea-hypopnea syndrome

**DOI:** 10.1007/s11325-024-03189-9

**Published:** 2025-01-08

**Authors:** Dóra Sulina, Szilvia Puskás, Mária Tünde Magyar, László Oláh, Norbert Kozák

**Affiliations:** 1 Faculty of Medicine, Department of Neurology, University of Debrecen, Moricz Zs. str. 22, Debrecen, H-4032 Hungary; 2 General Family Practice, Diósd, Hungary

**Keywords:** Severe OSAHS, Carotid artery intima-media thickness, CPAP, Early atherosclerosis

## Abstract

**Purpose:**

Obstructive sleep apnea-hypopnea syndrome (OSAHS) is the most common sleep-related breathing disorder. Longer term, repeated episodes of hypercapnia and hypoxemia during sleep are associated with inflammatory and atherosclerosis-related factors. The aim of this study was to explore the effect of continuous positive airway pressure (CPAP) therapy on cerebral vasoreactivity and early atherosclerosis in patients with severe OSAHS.

**Methods:**

Forty-one patients with severe OSAHS were enrolled. The mean follow-up time was 39.8 ± 9.1 months. Cardiovascular risk factors were assessed, and laboratory tests, carotid artery intima-media thickness (CIMT) measurement and cerebrovascular reserve capacity (CRC) measurement were performed. After the baseline examination, 28 patients received CPAP therapy (treated group), which was not available for 13 patients (untreated group). Parameters were compared before and after treatment, between treated and untreated patients.

**Results:**

Cardiovascular risk factors, baseline polysomnographic parameters, laboratory values, CIMT and CRC of the two groups were similar at baseline. At the follow-up, CRC did not differ between the two groups, but CIMT was significantly lower in the treated group than in the untreated group (0.73 ± 0.11 mm vs. 0.84 ± 0.21 mm, *p* = 0.027). The CIMT of both groups increased significantly during the follow-up period (from 0.65 ± 0.11 mm to 0.73 ± 0.11 mm in the treated group, and from 0.69 ± 0.11 mm to 0.84 ± 0.21 mm in the untreated group), but the increase in the treated group was smaller than in the untreated group (0.09 ± 0.09 mm vs. 0.15 ± 0.15 mm).

**Conclusion:**

In patients with severe OSAHS, CPAP treatment significantly reduced the progression of CIMT.

## Introduction

We spend almost one third of our lives sleeping, and sleep disorders are very common. Sleep problems are a rapidly growing area of interest due to their increasing prevalence and social consequences. According to numerous epidemiologic studies, 30% of the population suffers from insomnia, and the prevalence of sleep apnea (apnea-hypopnea index [AHI] > 5/h) is estimated to be 14% of the world’s population between the ages of 30 and 69 [1].

Obstructive sleep apnea-hypopnea syndrome (OSAHS) is the most common sleep-related breathing disorder and is caused by partial or total obstruction of the upper respiratory tract. The severity of the disease is determined by the number of total apneic and hypopneic events per hour of sleep (normal: AHI < 5/h) as determined by polysomnography (in the present study the American Academy of Sleep Medicine (AASM) 2012 criteria were used with at least 3% desaturation for hypopnoe).

Sleep apnea has many short- and long-term effects on quality of life and health. Daytime sleepiness, fatigue, decreased concentration and performance and impaired memory are associated with work-related human error and motor vehicle accidents. Approximately 20% of car accidents are due to driver fatigue caused by insufficient and inadequate sleep [2]. Longer term, repeated episodes of cyclic hypercapnia, hypoxemia, ventilatory effort and microarousals during sleep are associated with abnormal inflammatory and atherosclerosis-related factors (e.g. endothelin-1, NO) and lead to proven objective clinical consequences, such as hypertension [3], insulin resistance [4], cardiovascular disease [5], cardiac arrythmias and depression [6].

Carotid intima-media thickness (CIMT) is a widely used ultrasound marker of early atherosclerosis and subclinical organ damage. Measurement of CIMT is a noninvasive, reproducible, cost-effective, easily implementable method that is suitable for assessing the degree of progression of atherosclerosis [7]. Thickening of the internal carotid wall is a natural, dynamic, lifelong process. A cross-sectional study estimated that the average increase in CIMT is approximately 0.01 mm/year [8]. However, multiple cardiovascular risk factors affect CIMT (e.g., hypertension, diabetes, smoking, and high lipid levels), and patients suffering from OSAHS without a history or risk factors for cardiovascular disease have also been shown to have greater CIMT [9].

Cerebrovascular reactivity provides information about vasodilatory properties of the cerebral microvessels. As a result of hypoxia or hypercapnia, cerebral arterioles dilate, thus increasing the cerebral blood flow. Changes in cerebral blood flow in the area supplied by an artery can be assessed by monitoring changes in cerebral artery flow velocity using transcranial Doppler (TCD). Cerebrovascular reserve capacity (CRC) is defined as the maximum percentage increase in cerebral blood flow induced by a vasodilatory stimulus. The most common method of measuring CRC is the breath-holding test. Breath holding increases the partial pressure of carbon dioxide, which is a potent vasodilator of cerebral arterioles, but does not change or minimally changes the diameter of large intracranial vessels [10]. Vasodilation in the arterioles and cerebral microvessels results in a decrease in vascular resistance and consequently increases the flow and flow velocity in the large cerebral arteries including the middle cerebral artery (MCA). Severely damaged CRC (below 15%) has been shown to be a risk factor for stroke [11]. In obstructive sleep apnea, CRC has been shown to be reduced, i.e., the cerebral arterioles do not respond properly to various vasodilatory stimuli [12]. The reduced CRC together with the sudden changes in blood pressure due to sympathicotonia increases the cerebrovascular risk, because cerebral resistance vessels with reduced vasodilatory capacity are less able to adapt to the changing blood pressure [13].

Sleep apnea is a treatable disorder, and its first-line therapy is the use of continuous positive airway pressure (CPAP) device. Effective treatment prevents apnea and hypopnea, normalizes the number of microarousals.

Our study was conducted to determine whether CPAP therapy for more than one year can positively influence atherosclerosis and cerebral vasoreactivity in patients with severe OSAHS.

## Methods

The present study was conducted between April 14, 2011 and April 10, 2016 in the Sleep Laboratory of the Department of Neurology (University of Debrecen), which is accredited by the Hungarian Society for Sleep Medicine and the European Sleep Research Society. Only patients diagnosed with severe sleep apnea syndrome (OSAHS) between the ages of 18 and 80 years were included after providing signed informed consent. The clinical diagnosis of OSAHS was made by polysomnography evaluated by a qualified sleep physician (somnologist), and severe OSAHS was determined if the total number of apnea and hypopnea events per hour was at least 30 (AHI ≥ 30/h) in accordance with the American Academy of Sleep Medicine (2012 criteria, at least 3% desaturation for hypopnoe). Patients with severe heart disease (severe chronic heart failure (more than NYHA II) and significant valvular dysfunction), severe renal or hepatic failure, malignancy, autoimmune or chronic inflammatory disease were excluded.

Medical history, demographics, body mass index, and cardiovascular risk factors were assessed, and a detailed neurological physical examination, laboratory tests and measurements of carotid intima-media thickness and cerebrovascular reserve capacity were performed. CIMT was analyzed offline.

On the day of the baseline examination, the enrolled patients were not receiving CPAP therapy. After the baseline examination, continuous positive airway pressure (CPAP) was manually adjusted for each patient in the Sleep Laboratory, but only 28 patients received CPAP treatment; for financial reasons CPAP therapy was not available for 13 patients. After 27 to 59 months of follow-up, the baseline examinations were repeated in the treated and untreated groups.

The current study protocol was approved by the Regional Institutional Research Ethics Committee of the University of Debrecen, and informed consent was signed by all patients.

### Polysomnography

Overnight, in-lab diagnostic polysomnography (Philips Alice III and V) was performed by trained sleep technicians certified in clinical electrophysiology. Continuous recording of 4 EEG channels (EEG electrodes: F3-M2, C3-M2, C4-M1, O2-M1), 2 EOG channels, 1 submental EMG channel, 1 ECG channel, and additional channels from the nasal pressure/thermal flow sensor, pulse oximetry, thoracic and abdominal impedance belt, position sensor, and microphone provided real-time online data. Continuous supervision ensured the proper operation of the equipment and reduced the possibility of errors and malfunctions. Sleep stages and scoring were interpreted by a somnologist, and the severity of OSAHS was defined by the apnea-hypopnea index (mild: AHI: 5–14/h, moderate: AHI: 15–29/h, severe: AHI ≥ 30/h) (according to AASM 2012 criteria).

On the day of the PSG, the patients completed a questionnaire on cardiovascular risk factors, such as hypertension, diabetes mellitus, hyperlipidemia, smoking, alcohol consumption and history of cardiovascular events (myocardial infarction, angina pectoris and stroke). Laboratory tests were also performed on the same morning. Glucose, hemoglobin A1c (HgA1c), CRP, white blood cell (WBC), cholesterol (Chol) and high-density lipoprotein cholesterol (HDL-C) levels were measured.

### Measurement of carotid intima-media thickness

During CIMT measurement, patients were in the supine position with the neck turned to the opposite side of the measurement. Assessment was performed with a high-resolution ultrasound device (Philips HD11, Philips, Amsterdam, The Netherlands), which included a 3-lead ECG system. On B-mode images, longitudinal ultrasound images of the common carotid artery and carotid bifurcation were visualized with a linear transducer (5,5–7,5 Mhz). Measurements were taken on the far wall of the common carotid artery at least 10 mm far from its distal end, where the vessel was free of atherosclerotic plaque and the lumen-intima and media-adventitia boundaries were clearly visible. The average of 10 measurements taken offline on each side during the systolic phase provided the right and left intima-media thickness values (mm). The mean CIMT value was calculated as the mean of the left and right CIMT (mm) [14].

### Cerebrovascular reserve capacity

The breath-hold test was used to assess the cerebrovascular reserve capacity (CRC) in the MCAs on both sides. Patients were placed in the supine position and breathed normally. MCA flow velocity was assessed by transcranial Doppler (Multidop T, DWL, Singen, Germany)) equipped with a handheld 2 MHz pulse-wave probe. The measurement was performed through the transtemporal window at a depth of 50–55 mm. Before the breath-hold test, the MCA mean blood flow velocity was monitored for 5 min during normal breathing to obtain the baseline mean blood flow velocity data (MBFVbefore). Then, after a normal inspiration, the patients were asked to hold their breath for at least 30 s, without performing the Valsalva maneuver. At the end of the breath-holding period, the maximum value of the middle cerebral artery MBFV (MBFVmax) was recorded. The breath-holding index was calculated as the ratio of the percentage change in velocity to the duration of the breath-holding (time): [(MBFVmax – MBFVbefore)/time] × 100 [15].

Laboratory tests, CIMT and CRC measurements were performed at baseline and at the end of an average follow-up of 39 months. The parameters of treated (*n* = 28) and untreated patients (*n* = 13) were compared, as well as the parameters of treated patients before and after CPAP treatment (*n* = 28).

### Statistical analysis

SPSS software was used for the statistical analysis. In addition to descriptive statistics, the Pearson χ2 test was used for categorical variables. After testing for normal distribution, paired t-tests were used to compare data before and after CPAP treatment, and unpaired t-tests were used to compare data from the treated and untreated groups. A value of *p* < 0.05 was considered significant.

## Results

We performed a follow-up study of forty-one patients with severe OSAHS (mean AHI: 60.5/h ± 16.1; mean desaturation index [DI]: 45.8/h ± 23.4; mean age 53.9 ± 10.2 years). The mean follow-up time was 39.8 ± 9.1 months.

The mean age, risk factors, baseline PSG parameters, BMI, laboratory values, CIMT and CRC and the follow-up time of the treated and untreated groups were similar [Table 1].

**Table 1:** Baseline characteristics of patients at enrollment

	Untreated (*n* = 13)	Treated(*n* = 28)	*p* value
Age, years	57.54 ± 9.17	52.29 ± 10.34	0.125
Sex (male), n	10 (77%)	25 (89.3%)	0.297
Smoking, n	3 (23%)	12 (43%)	0.221
Hypertension, n	9 (69%)	23 (82%)	0.352
AHI, events/h	56.67 ± 15.19	62.30 ± 16.43	0.302
BMI, kg/m²	36.04 ± 5.65	37.20 ± 6.70	0.594
Average CIMT, mm	0.69 ± 0.11	0.65 ± 0.11	0.216
CRC, %	51.76 ± 14.58	45.61 ± 14.11	0.263
glucose, (mmol/l)	6.31 ± 0.86	6.50 ± 2.18	0.762
HgA1C (%)	6.12 ± 0.79	6.33 ± 0.98	0.505
CRP (mg/l)	3.97 ± 2.99	6.07 ± 5.18	0.183
Chol (mmol/l)	5.22 ± 1.01	5.09 ± 1.02	0.687
HDL-C (mmol/l)	1.27 ± 0.32	1.14 ± 0.31	0.221

AHI, apnea-hypopnea index; BMI, body mass index; CIMT, carotid intima-media thickness; CRC, cerebrovascular reserve capacity; HgA1C, glycated hemoglobin; CRP, C-reactive protein; Chol, total cholesterol; HDL-C, high-density lipoprotein

PSG values (AHI, DI) were significantly better after CPAP treatment in the treated group than in the untreated group, indicating that the CPAP setting was appropriate [Table 2].

**Table 2:** PSG values at follow-up examination

	Untreated (*n* = 13)	Treated (*n* = 28)	*p* value
AHI, events/h	48.22 ± 15.88	1.97 ± 1.91	*p* < 0.001
DI, events/h	25.65 ± 10.47	1.62 ± 1.36	*p* < 0.001

DI, desaturation index

**Fig. 1:**
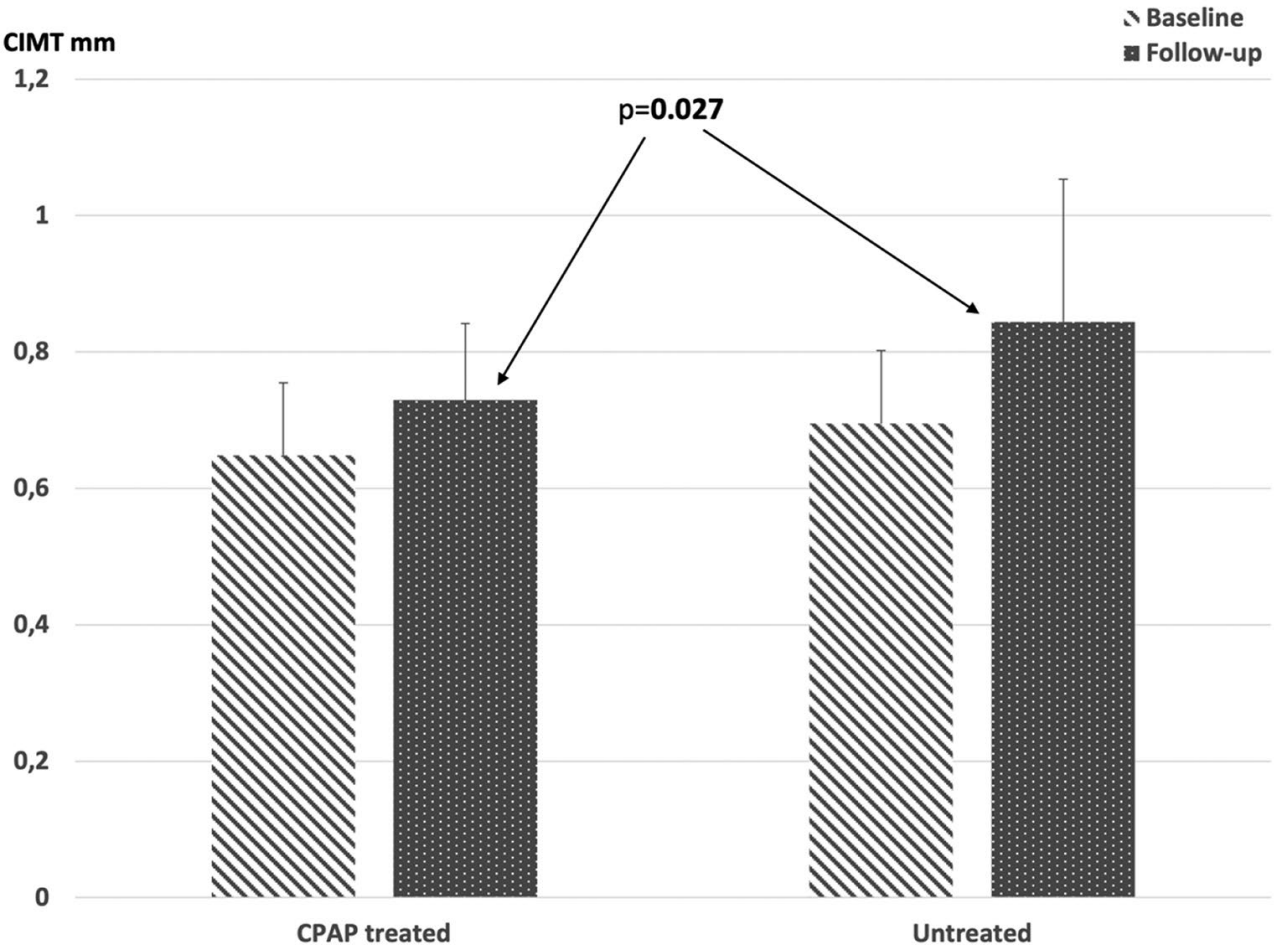
Comparison of the CIMT measured at the beginning of the study (baseline) and at the end of the follow-up examinations (follow-up) in treated and untreated patients

At the follow-up, BMI, laboratory values, and CRC did not differ between the treated and untreated groups [Table 3], but CIMT was significantly lower in the treated group than in the untreated group [Table 3; Fig. 1.]. The duration of follow-up periods in the treated and untreated groups was similar.

**Table 3:** Characteristics of the patients at the end of the follow-up period

	Untreated (*n* = 13)	Treated (*n* = 28)	*p* value
BMI, kg/m²	36.08 ± 5.65	36.62 ± 7.32	0.817
Average CIMT, mm	0.84 ± 0.21	0.73 ± 0.11	0.027
CRC, %	39.95 ± 14.22	47.3 ± 15.78	0.195
glucose, (mmol/l)	6.22 ± 0.93	6.03 ± 1.17	0.615
HgA1C (%)	6.15 ± 0.72	6.09 ± 0.98	0.825
CRP (mg/l)	4.46 ± 2.7	5.12 ± 7.52	0.762
Chol (mmol/l)	5.44 ± 0.91	5.16 ± 1.04	0.415
HDL-C (mmol/l)	1.24 ± 0.42	1.13 ± 0.27	0.351
Follow-up time (month)	40.92 ± 9.65	39.36 ± 9.06	0.617

**Fig. 2:**
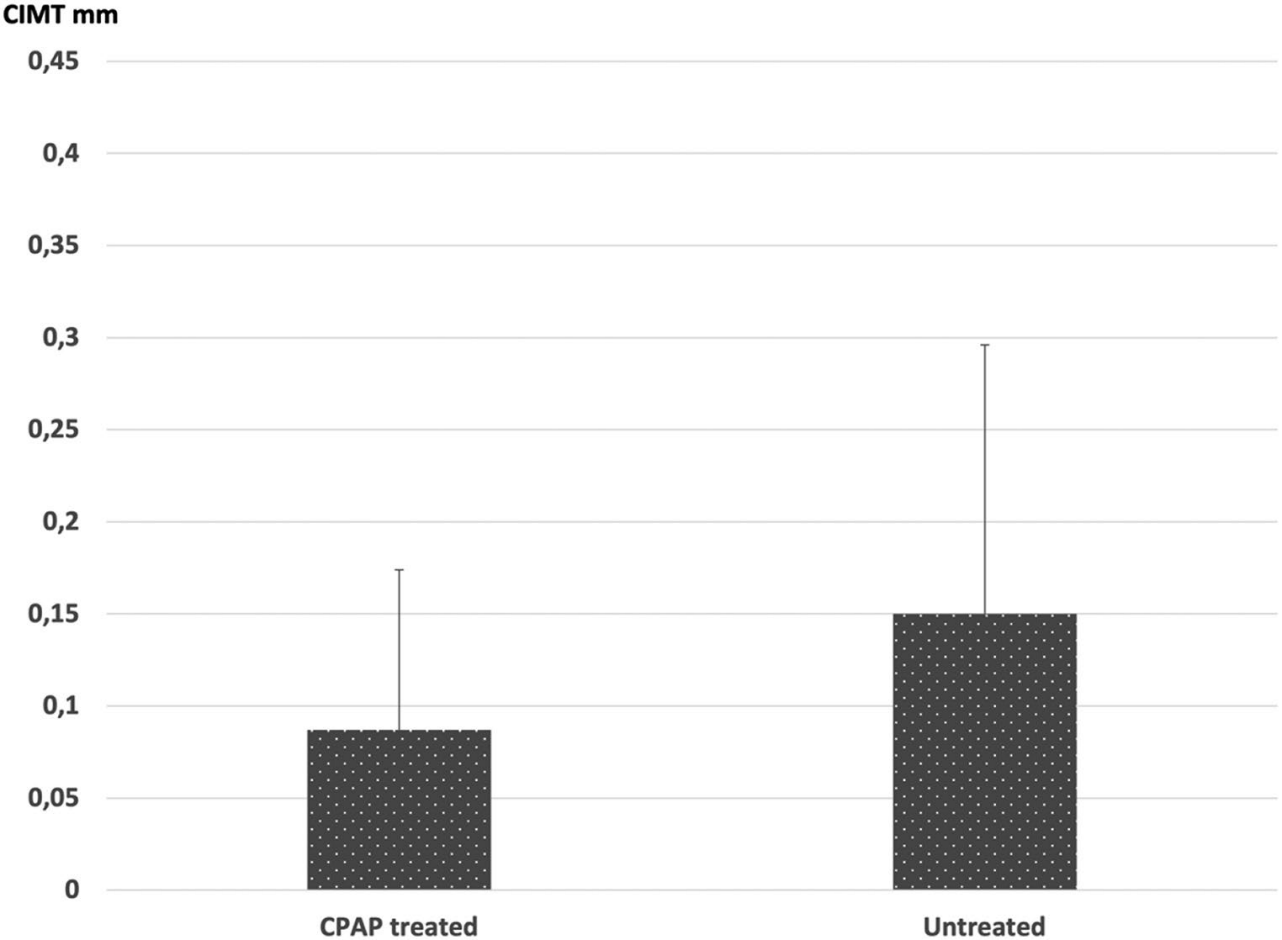
Increase in the CIMT during the follow-up period in the treated and untreated groups

CIMT increased significantly in both the treated and untreated groups during the follow-up period (from 0.64 ± 0.11 mm to 0.73 ± 0.11 mm in the treated group, *p* < 0.001, and from 0.69 ± 0.11 mm to 0.84 ± 0.21 mm in the untreated group, *p* = 0.004), but the increase in CIMT was less in the treated group than in the untreated group (0.09 ± 0.09 mm vs. 0.15 ± 0.15 mm; *p* = 0.101) (Fig. 2).

## Discussion

In recent years, increasing attention has been paid to understanding the background of cardiovascular events and better assessing the relationship between OSAHS and cerebrovascular and cardiovascular disease. OSAHS is a non-invasively diagnosed, easily treatable and common cardiovascular risk factor.

It is well known that about 50% of patients with OSAHS have hypertension, and it has been shown that almost 30% of patients with hypertension have OSAHS. One of the possible reasons for the association between OSAHS and hypertension is the apnea-induced hypoxia, which leads to nocturnal hypertension through sympathetic activation. The oxidative stress, systemic inflammation and endothelial dysfunction observed in OSAHS may also contribute to the development of hypertension [4]. Positive airway pressure therapy is known to reduce the sympathetic activity, leading to decrease in blood pressure. According to scientific reports, adequate CPAP treatment in OSAHS patients reduced systolic blood pressure by 3 mmHg and diastolic blood pressure by 2 mmHg [16].

In OSAHS patients, hypoxia was reported to be associated not only with increased blood pressure, but also with elevated serum catecholamine levels, metabolic dysregulation and pancreatic beta-cell dysfunction, resulting in reduced insulin resistance [17]. In addition to hypertension, well-adjusted CPAP treatment also improved metabolic parameters, including triglyceride, cholesterol and HgA1c levels [18, 19]. These data demonstrated that properly adjusted positive airway pressure therapy in OSAHS patients had a beneficial effect on most conventional vascular risk factors, including hypertension, hyperlipidemia and diabetes mellitus.

Consistent with literature data, hypertension and diabetes mellitus were more common in patients with severe OSAHS in the present study (hypertension 78.05%, diabetes mellitus 21.95%) than in the average Hungarian population (hypertension 32%, diabetes mellitus 14%) [20].

Bessler et al. found that repeated hypoxia and hypercapnia in patients with sleep apnea syndrome caused an increased levels of inflammatory markers such as C-reactive protein (CRP), tumor necrosis factor (TNF-α) and interleukin-6 (IL- 6) through the oxidative stress [21]. Although the CRP level in our patients was at the upper limit of the reference value, there was no significant difference between the CRP measured at baseline and after CPAP therapy.

One of the long-term consequences of OSAHS is the increased cardiovascular risk. Therefore, CIMT measurement, which can be used to detect early atherosclerosis, is extremely important in OSAHS patients. Investigation of CIMT and serum levels of inflammatory markers in 36 patients with OSAHS and 16 obese control subjects [22] showed significantly larger CIMT, serum CRP, interleukin-6 and interleukin-8 levels in the OSAHS group compared with the obese control group. CIMT was significantly correlated with the serum levels of CRP, interleukin-6, and interleukin-8, as well as with the duration of hypoxia and the severity of OSAHS. Another previous study also reported that CIMT was greater in patients with obstructive sleep apnea than in healthy controls, and the presence of atherosclerotic plaques and stenosis of the common carotid artery was more prevalent in OSAHS patients than in the controls [23]. These results support that hypoxia and systemic inflammation associated with OSAHS promote the progression of atherosclerosis and that OSAHS has a direct effect on the development of early atherosclerosis, increasing the cardiovascular and cerebrovascular morbidity in these patients. Several studies have demonstrated that OSAHS is an independent risk factor for CIMT, which has been shown to be a suitable marker for monitoring and determining the severity of early atherosclerosis in OSAHS patients [24].

To the best of our knowledge, only few studies with 1-year follow-up have investigated the effect of CPAP treatment on CIMT in OSAHS patients, and the results are conflicting. A meta-analysis showed that CPAP had no effect on CIMT in OSAHS patients, although CIMT was significantly reduced after CPAP therapy in more severe OSAHS patients and those with longer CPAP use [25]. Contrary to this meta-analysis, a prospective cohort study with a one-year follow-up showed that CIMT decreased significantly in OSAHS patients treated with CPAP, but increased in patients receiving conservative medical therapy without CPAP treatment [26]. Subsequent studies demonstrated an association between OSAHS severity and CIMT [26 27], as well as between AHI and CIMT [28]. After 6 months of CPAP treatment, CIMT decreased in both studies [27, 28]. According to our study, the increase of CIMT was significantly slower in patients using CPAP, suggesting that CPAP reduces the progression of early atherosclerosis in OSAHS patients.

In view of the fact that a previous study has confirmed reduced cerebrovascular reserve capacity in patients with OSAHS [29], we performed CRC measurements in all patients in addition to CIMT measurements. The CRC did not change significantly in our study, but it did not progress either. Placidi et al. suggested that cerebrovascular chemoreceptors in patients with obstructive sleep apnea syndrome become hyposensitive due to the stress caused by continuous nocturnal hypercapnia. In contrast to our results, Piraino et al. found that the BHI increased after CPAP therapy in moderate to severe OSAHS patients [30], suggesting that long-term CPAP treatment may improve the cerebral vascular regulation and reduce the risk of ischemic stroke in patients with obstructive sleep apnea syndrome.

The present study has several notable strengths. The follow-up time was more than 3 years, and we were able to enroll and follow untreated patients with severe OSAHS. However, there are some limitations that need to be considered. First, the sample size was relatively small, although the effect of CPAP treatment on CIMT was still demonstrated. In the future, a larger sample size is needed to better understand the effect of CPAP treatment on cerebrovascular risk factors. Second, the poor financial situation in the untreated group may be related to worse health outcomes which may also affect CIMT. However, there were no significant differences in the cardiovascular parameters and risk factors between the two groups except for CIMT and PSG parameters at follow-up.

## Conclusions

CPAP treatment significantly slowed the progression of CIMT in patients with severe OSAHS, but no significant changes in cerebrovascular reserve capacity were found after long-term CPAP treatment. These data suggest that CPAP therapy reduces the progression of early atherosclerosis in patients with sleep apnea syndrome and may thus contribute to stroke prevention.

## Data Availability

The data that support the findings of this study are available from the authors.

## References

[1] Young T, Palta M, Dempsey J, Skatrud J, Weber S, Badr S (1993) The occurrence of sleep-disordered breathing among middle-aged adults. N Engl J Med.; 328(17):1230-5. 10.1056/NEJM199304293281704. PMID: 8464434 10.1056/NEJM199304293281704 8464434

[2] de Mello MT, Narciso FV, Tufik S, Paiva T, Spence DW, Bahammam AS, Verster JC, Pandi-Perumal SR (2013) Sleep disorders as a cause of motor vehicle collisions. Int J Prev Med 4(3):246–257 PMID: 23626880; PMCID: PMC3634162 23626880 PMC3634162

[3] Mohsenin V (2014) Obstructive sleep apnea and hypertension: a critical review. Curr Hypertens Rep 16:482. 10.1007/s11906-014-0482-4 25139780 10.1007/s11906-014-0482-4

[4] Bruyneel M, Kleynen P, Poppe K (2020) Prevalence of undiagnosed glucose intolerance and type 2 diabetes in patients with moderate-to-severe obstructive sleep apnea syndrome. Sleep Breath 24(4):1389–1395. 10.1007/s11325-019-01989-yEpub 2019 Dec 14. PMID: 31838624 31838624 10.1007/s11325-019-01989-y

[5] Hla KM, Young T, Hagen EW, Stein JH, Finn LA, Nieto FJ, Peppard PE (2015) Coronary heart disease incidence in sleep disordered breathing: the Wisconsin Sleep Cohort Study. Sleep 38(5):677–684. 10.5665/sleep.4654PMID: 25515104; PMCID: PMC4402672 25515104 10.5665/sleep.4654 PMC4402672

[6] Acker J, Richter K, Piehl A, Herold J, Ficker JH, Niklewski G (2017) Obstructive sleep apnea (OSA) and clinical depression-prevalence in a sleep center. Sleep Breath.; 21(2):311–318. 10.1007/s11325-016-1411-3. Epub 2016 Oct 5. PMID: 27704327 10.1007/s11325-016-1411-3 27704327

[7] Grobbee DE, Bots ML (1994) Carotid artery intima-media thickness as an indicator of generalized atherosclerosis. J Intern Med. 236(5):567 – 73. 10.1111/j.1365-2796.1994.tb00847.x. PMID: 7964435 10.1111/j.1365-2796.1994.tb00847.x 7964435

[8] Howard G, Sharrett AR, Heiss G, Evans GW, Chambless LE, Riley WA, Burke GL (1993) Carotid artery intimal-medial thickness distribution in general populations as evaluated by B-mode ultrasound. ARIC Investigators. Stroke. 24(9):1297 – 304. 10.1161/01.str.24.9.1297. PMID: 8362421 10.1161/01.str.24.9.1297 8362421

[9] Fox N, Ayas N, Park JE, Fleetham J, Frank Ryan C, Lear SA, Mulgrew A, Chan S, Hill J, John Mancini GB, Wong GC (2014) Carotid intima media thickness in patients with obstructive sleep apnea: comparison with a community-based cohort. Lung. 192(2):297–303. 10.1007/s00408-014-9556-y. Epub 2014 Jan 29. PMID: 24469113 10.1007/s00408-014-9556-y 24469113

[10] Djurberg HG, Seed RF, Evans DA, Brohi FA, Pyper DL, Tjan GT, alMoutaery KR (1998) Lack of effect of CO 2 on cerebral arterial diameter in man. J Clin Anesth 10:646–651 9873965 10.1016/s0952-8180(98)00107-x

[11] Gur AY, Bova I, Bornstein NM (1996) Is impaired cerebral vasomotor reactivity a predictive factor of stroke in asymptomatic patients? Stroke. 27(12):2188-90. 10.1161/01.str.27.12.2188. PMID: 8969778 10.1161/01.str.27.12.2188 8969778

[12] Furtner M, Staudacher M, Frauscher B, Brandauer E, Esnaola y Rojas MM, Gschliesser V, Poewe W, Schmidauer C, Ritsch-Marte M, Högl B (2009) Cerebral vasoreactivity decreases overnight in severe obstructive sleep apnea syndrome: a study of cerebral hemodynamics. Sleep Med. 10(8):875 – 81. doi: 10.1016/j.sleep.2008.09.011. Epub 2009 Feb 5. PMID: 19200779 10.1016/j.sleep.2008.09.011 19200779

[13] Hermann DM, Bassetti CL (2003) Sleep-disordered breathing and stroke. Curr Opin Neurol. 16(1):87–90. 10.1097/01.wco.0000053587.70044.be. PMID: 12544862 10.1097/01.wco.0000053587.70044.be 12544862

[14] Touboul PJ, Hennerici MG, Meairs S, Adams H, Amarenco P, Bornstein N, Csiba L, Desvarieux M, Ebrahim S, Hernandez Hernandez R, Jaff M, Kownator S, Naqvi T, Prati P, Rundek T, Sitzer M, Schminke U, Tardif JC, Taylor A, Vicaut E, Woo KS (2012) Mannheim carotid intima-media thickness and plaque consensus (2004-2006-2011). An update on behalf of the advisory board of the 3rd, 4th and 5th watching the risk symposia, at the 13th, 15th and 20th European Stroke Conferences, Mannheim, Germany, 2004, Brussels, Belgium, 2006, and Hamburg, Germany, 2011. Cerebrovasc Dis. 34(4):290-6. 10.1159/000343145. Epub 2012 Nov 1. PMID: 23128470; PMCID: PMC3760791 10.1159/000343145 PMC3760791 23128470

[15] Zavoreo I, Demarin V (2004) Breath Holding Index in the Evaluation of Cerebral Vasoreactivity. *Acta clinica Croatica, 43* (1), 15–19. Retrieved from https://hrcak.srce.hr/14437

[16] Alajmi M, Mulgrew AT, Fox J et al (2007) Impact of continuous positive Airway pressure therapy on blood pressure in patients with obstructive sleep apnea hypopnea: a Meta-analysis of Randomized controlled trials. Lung 185:67–72. 10.1007/s00408-006-0117-x 17393240 10.1007/s00408-006-0117-x

[17] Wilcox I, McNamara SG, Collins FL, Grunstein RR, Sullivan CE (1998) Syndrome Z: the interaction of sleep apnoea, vascular risk factors and heart disease. Thorax 53(Suppl 3):S25–S28 PMID: 10193357; PMCID: PMC1765908 10193357 PMC1765908

[18] Kostopoulos K, Alhanatis E, Pampoukas K et al (2016) CPAP therapy induces favorable short-term changes in epicardial fat thickness and vascular and metabolic markers in apparently healthy subjects with obstructive sleep apnea-hypopnea syndrome (OSAHS). Sleep Breath 20:483–493. 10.1007/s11325-015-1236-5 26223484 10.1007/s11325-015-1236-5

[19] Herth J, Sievi NA, Schmidt F, Kohler M (2023) Effects of continuous positive airway pressure therapy on glucose metabolism in patients with obstructive sleep apnoea and type 2 diabetes: a systematic review and meta-analysis. Eur Respir Rev 32(169):230083 PMID: 37673425; PMCID: PMC10481331 37673425 10.1183/16000617.0083-2023 PMC10481331

[20] Központi Statisztikai Hivatal (2021) Egészségügyi helyzetkép, 2019. Központi Statisztikai Hivatal. https://www.ksh.hu/apps/shop.kiadvany?p_kiadvany_id=1063993%26p_temakor_kod=KSH%26p_lang=HU [in hungarian]

[21] Baessler A, Nadeem R, Harvey M, Madbouly E, Younus A, Sajid H, Naseem J, Asif A, Bawaadam H (2013) Treatment for sleep apnea by continuous positive airway pressure improves levels of inflammatory markers - a meta-analysis. J Inflamm (Lond) 10:13. 10.1186/1476-9255-10-13PMID: 23518041; PMCID: PMC3637233 23518041 10.1186/1476-9255-10-13 PMC3637233

[22] Minoguchi K, Yokoe T, Tazaki T, Minoguchi H, Tanaka A, Oda N, Okada S, Ohta S, Naito H, Adachi M (2005) Increased carotid intima-media thickness and serum inflammatory markers in obstructive sleep apnea. Am J Respir Crit Care Med. 172(5):625 – 30. 10.1164/rccm.200412-1652OC. PMID: 16120716 10.1164/rccm.200412-1652OC 16120716

[23] Schulz R, Seeger W, Fegbeutel C, Hüsken H, Bödeker RH, Tillmanns H, Grebe M (2005) Changes in extracranial arteries in obstructive sleep apnoea. Eur Respir J. 25(1):69–74. 10.1183/09031936.04.00030004. PMID: 15640325 10.1183/09031936.04.00030004 15640325

[24] Zhou M, Guo B, Wang Y, Yan D, Lin C, Shi Z (2017) The Association between Obstructive Sleep Apnea and Carotid Intima-Media thickness: a systematic review and Meta-analysis. Angiology 68(7):575–583 Epub 2016 Aug 31. PMID: 27581069 27581069 10.1177/0003319716665985

[25] Chen LD, Lin L, Lin XJ, Ou YW, Wu Z, Ye YM, Xu QZ, Huang YP, Cai ZM (2017) Effect of continuous positive airway pressure on carotid intima-media thickness in patients with obstructive sleep apnea: a meta-analysis. PLoS ONE 12(9):e0184293. 10.1371/journal.pone.0184293PMID: 28863162; PMCID: PMC5580911) 28863162 10.1371/journal.pone.0184293 PMC5580911

[26] Hui DS, Shang Q, Ko FW, Ng SS, Szeto CC, Ngai J, Tung AH, To KW, Chan TO, Yu CM (2012) A prospective cohort study of the long-term effects of CPAP on carotid artery intima-media thickness in obstructive sleep apnea syndrome. Respir Res 13(1):22. 10.1186/1465-9921-13-22PMID: 22424053; PMCID: PMC3337823 22424053 10.1186/1465-9921-13-22 PMC3337823

[27] El-Azem I, Amal abd, Mohamed MN, AboZaid, Hosam M (2018) Attia the effect of treatment with continuous positive airway pressure on carotid artery intima-media thichness and the correlation between obstructive sleep apnea severity and carotid wall thichness in OSAS patients. Egyiptian J Chest Dis Tuberculosis 67:453–458

[28] Jiang YQ, Xue JS, Xu J, Zhou ZX, Ji YL (2017) Efficacy of continuous positive airway pressure treatment in treating obstructive sleep apnea hypopnea syndrome associated with carotid arteriosclerosis. Exp Ther Med 14(6):6176–6182. 10.3892/etm.2017.5308Epub 2017 Oct 16. PMID: 29285176; PMCID: PMC5740802 29285176 10.3892/etm.2017.5308 PMC5740802

[29] Placidi F, Diomedi M, Cupini LM, Bernardi G, Silvestrini M (1998) Impairment of daytime cerebrovascular reactivity in patients with obstructive sleep apnoea syndrome. J Sleep Res. 7(4):288 – 92. 10.1046/j.1365-2869.1998.00120.x. PMID: 9844856 10.1046/j.1365-2869.1998.00120.x 9844856

[30] Piraino A, Sette G, D’Ascanio M, La Starza S, Aquilini M, Ricci A (2019) Effect of OSAS on cerebral vasoreactivity and cIMT before and after CPAP treatment. Clin Respir J 13(9):555–559. 10.1111/crj.13057Epub 2019 Jul 24. PMID: 31301263 31301263 10.1111/crj.13057

